# Targeting EGFR with photodynamic therapy in combination with Erbitux enhances in vivo bladder tumor response

**DOI:** 10.1186/1476-4598-8-94

**Published:** 2009-11-02

**Authors:** Ramaswamy Bhuvaneswari, Yik Yuen Gan, Khee Chee Soo, Malini Olivo

**Affiliations:** 1 National Cancer Centre Singapore, 11 Hospital Drive, 169610, Singapore; 2 Natural Sciences and Science Education, National Institute of Education, Nanyang Technological University, 1 Nanyang Walk, 637616, Singapore; 3 Singapore Bioimaging Consortium, Biomedical Sciences Institutes, 11 Biopolis Way, #02-02 Helios, 138667, Singapore; 4 Department of Physics, National University of Ireland, Galway, Ireland

## Abstract

**Background:**

Photodynamic therapy (PDT) is a promising cancer treatment modality that involves the interaction of the photosensitizer, molecular oxygen and light of specific wavelength to destroy tumor cells. Treatment induced hypoxia is one of the main side effects of PDT and efforts are underway to optimize PDT protocols for improved efficacy. The aim of this study was to investigate the anti-tumor effects of PDT plus Erbitux, an angiogenesis inhibitor that targets epidermal growth factor receptor (EGFR), on human bladder cancer model. Tumor-bearing nude mice were assigned to four groups that included control, PDT, Erbitux and PDT plus Erbitux and tumor volume was charted over 90-day period.

**Results:**

Our results demonstrate that combination of Erbitux with PDT strongly inhibits tumor growth in the bladder tumor xenograft model when compared to the other groups. Downregulation of EGFR was detected using immunohistochemistry, immunofluorescence and western blotting. Increased apoptosis was associated with tumor inhibition in the combination therapy group. In addition, we identified the dephosphorylation of ErbB4 at tyrosine 1284 site to play a major role in tumor inhibition. Also, at the RNA level downregulation of EGFR target genes cyclin D1 and c-myc was observed in tumors treated with PDT plus Erbitux.

**Conclusion:**

The combination therapy of PDT and Erbitux effectively inhibits tumor growth and is a promising therapeutic approach in the treatment of bladder tumors.

## Background

Photodynamic therapy (PDT) is a treatment modality that involves the administration of a tumor-localizing photosensitizer followed by light irradiation of specific wavelength that matches the absorption characteristics of the photosensitizer, thereby producing cytotoxic intermediates that damage cellular structures [1]. The advantages of PDT include selective targeting, minimal invasiveness and reduced toxicity that allows for repeated treatment [2,3]. However during PDT, tumor oxygen is depleted due to vascular damage and oxygen consumption, which causes hypoxia within the surviving tumor cells thus triggering angiogenesis [4,5]. Angiogenesis is the sprouting of new smaller vessels from the pre-existing vasculature. Not only is angiogenesis essential for tumor growth but it also enables the migration of tumor cells to distant sites, forming metastases [6].

Bladder cancer is the 9^th ^most common cancer affecting Singapore men [7]. Current treatment options include surgery, chemotherapy or immunotherapy, and radiation therapy [8]. Efforts are on going to develop therapeutic tools that allow the preservation of bladder and to control the rate of recurrences. Clinical trials with PDT have shown promising results in the treatment of bladder cancer, especially for flat malignant lesions such as carcinoma in situ [9,10]. Recently, significant progress has also been made to understand the molecular and genetic events underlying bladder cancer [11]. Epidermal growth factor receptor (EGFR) is one such molecular marker that has been widely reported in bladder carcinoma [12,13]. Upregulated EGFR signaling is known to initiate a cascade of events leading to cell proliferation, migration, invasion [14] and blocking of apoptosis [15] that eventually leads to tumor progression. Many epithelial cancers have been found to overexpress EGFR, including head and neck, breast, colon, lung, prostate, kidney and bladder [16]. Studies show that antibodies that block the EGF binding site of EGFR inhibit tumor cell proliferation [17]. Therefore, blocking EGFR along with conventional cancer therapies could be an attractive anti-tumor strategy.

Erbitux (cetuximab), a chimeric human-murine monoclonal antibody, competitively binds to the accessible extracellular domain of EGFR and inhibits dimerisation and subsequently inhibits cell proliferation, tumor growth and metastasis [18]. In most studies, the use of Erbitux, as an anti-EGFR therapy in combination with chemotherapy and radiotherapy has demonstrated significant clinical efficacy, due to its good tolerability and non-overlapping toxicities [19]. Also, in vivo therapies with Erbitux and chemotherapy drugs resulted in a greater regression of bladder tumor growth compared with either agent alone [20]. In the present study we have evaluated the anti-tumor effect of Erbitux in combination with PDT on bladder carcinoma xenograft model. Our findings indicate that combining PDT and Erbitux significantly enhances the anti-tumor activity, by inhibiting EGFR expression, increasing apoptosis and by dephosphorylating essential EGFR tyrosine sites. These results may provide a rationale for evaluating the combination of PDT and Erbitux as a cancer treatment modality in a clinical setting.

## Results

### Tumor regression

**Figure 1:**
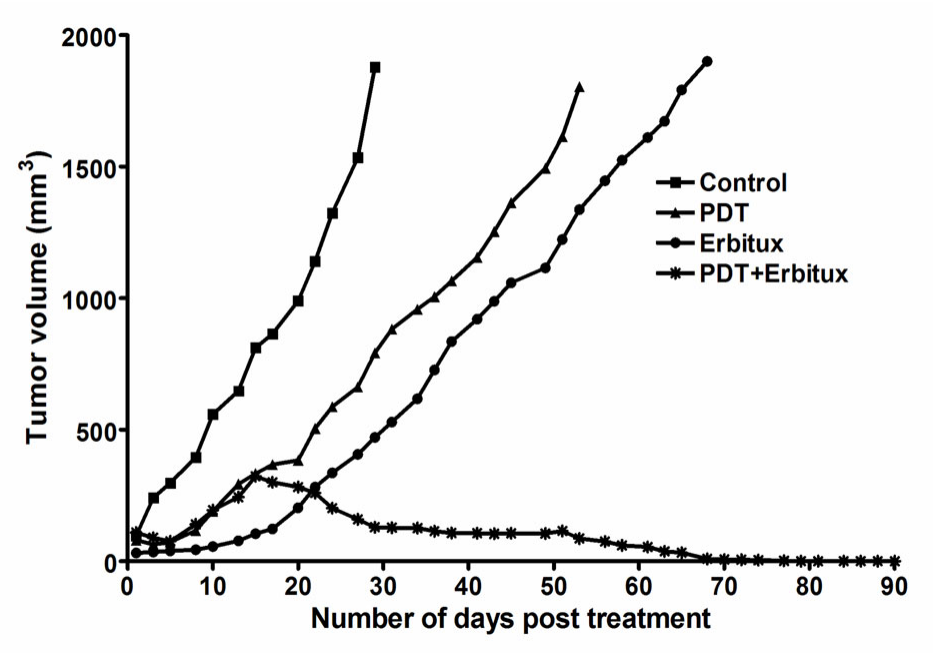
**Mean tumor volume charted against number of days post treatment, to assess the tumor response in various treatment groups**. The combination therapy group of PDT and Erbitux exhibited greatest tumor response in comparison with all other groups. Each group represents the mean response (bars, SE) of 10 animals.

To investigate the long-term effectiveness of PDT and Erbitux, we employed MGH bladder tumor xenograft model in athymic nude mice. Tumors were allowed to grow to sizes of 6-7 mm in diameter before PDT treatment was carried out and were measured three times a week and charted for 90 days (Figure 1). The total tumor volume for each group equals the sum of individual tumor volumes, which in our case were 8-10 individual tumors. Tumor inhibition was calculated on day 29 when the control tumors reached maximum volume of 2 cm^3^. The mean relative tumor inhibition of 93% (95% CI - 87.7 to 98) (*p *< 0.001) was observed in tumors treated with the combination therapy of PDT plus Erbitux when compared with control tumors. A week after treatment, accelerated tumor growth was noticed in the combination therapy group, but there was a decrease thereafter in tumor size, resulting in complete tumor regression. The tumors treated with PDT only and Erbitux only, exhibited 57.8% (95% CI - 49.2 to 66.4) and 74.8% (95% CI - 68.7 to 80.8) mean tumor inhibition respectively. Compared to control, the overall tumor response was greater in the monotherapy groups of PDT only and Erbitux only, though the difference between the monotherapy groups were not significant. The treatment modalities in our study did not induce any signs of toxicity such as excessive weight loss, diarrhea or vomiting in the animals. No treatment-related death occurred.

### Detection of EGFR in tumor tissue

**Figure 2:**
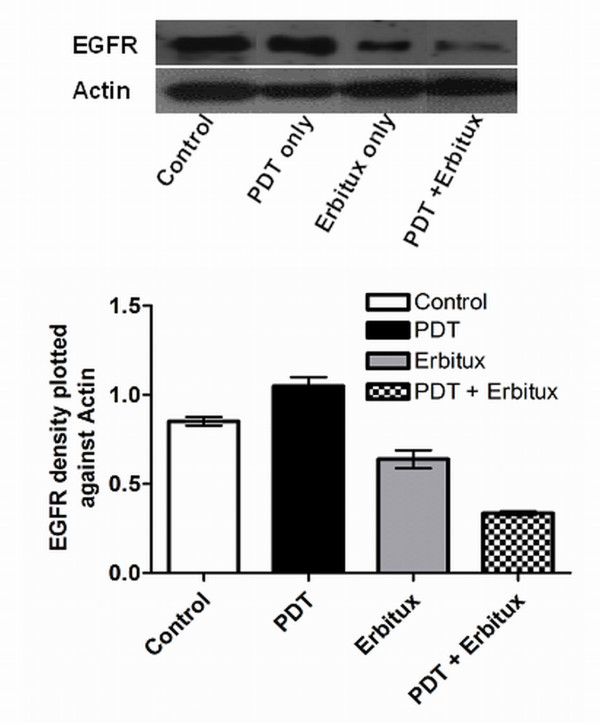
**Expression of EGFR in the tumors was detected using western immunoblot analysis**. Expression of actin was used to monitor protein loading. Ratio of EGFR density plotted against actin. Combination modality of PDT plus Erbitux was effective in reducing the expression of EGFR in the tumor tissue.

**Figure 3:**
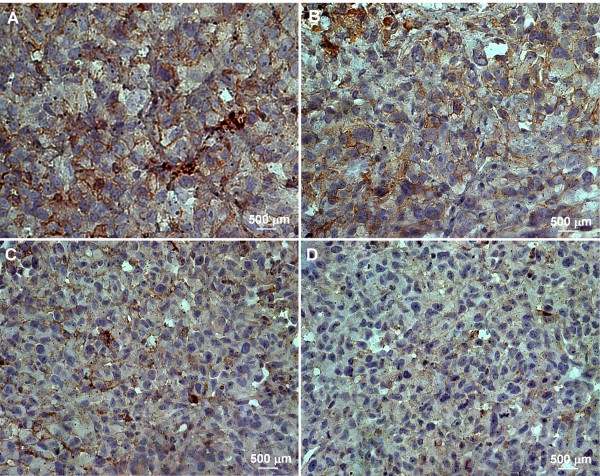
**EGFR expression was assessed in tumor sections using immunohistochemistry**. The brown colored membrane staining indicates EGFR positive immunoreactivity. (A: Control, B: PDT, C: Erbitux and D: PDT +Erbitux). Magnification: 630×.

**Figure 4:**
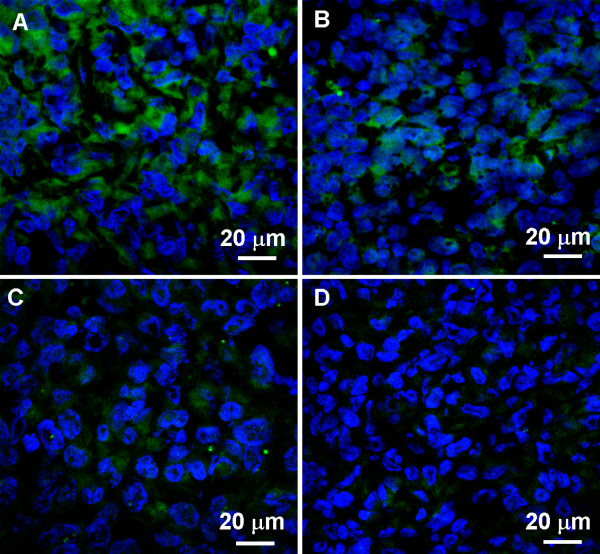
**Immunofluorescence was performed to confirm the above results**. In the confocal images, the green FITC fluorescence staining indicates the expression of EGFR. PDT and Erbitux (D) resulted in significant reduction of EGFR expression of 4-6% (EGFR score 1) compared to monotherapy (B: PDT and C: Erbitux) and control groups (A). Maximum EGFR tumor cell membrane staining of 21-24% (EGFR score 3) noticed in the untreated tumors. The monotherapy groups of PDT only and Erbitux only, exhibited 15-17% (EGFR score 2) and 11-13% (EGFR score 2) staining respectively. Magnification: 400×.

To investigate the anti-tumor activity of the treatments, EGFR expression was evaluated using western blotting. The results obtained were confirmed by immunohistochemistry (IHC) and immunofluorescence (IF) techniques. Tumors were harvested from the animals between 25-90 days, based on the maximum tumor volume limit or the completion of treatment. EGFR expression analyzed using immunoblotting was found to be lower in the PDT plus Erbitux group (*p *< 0.001) compared to control, PDT only and Erbitux only groups (Figure 2). IHC and IF results showed similar trends in which the combination of PDT and Erbitux resulted in significant reduction of EGFR expression at 4-6% (EGFR score 1) compared to monotherapy and control groups. Maximum EGFR tumor cell membrane staining of 21-24% (EGFR score 3) was noticed in the untreated tumors. The monotherapy groups of PDT only and Erbitux only, exhibited 15-17% (EGFR score 2) and 11-13% (EGFR score 2) staining respectively (Figures 3 and 4).

### Determination of apoptosis

**Figure 5:**
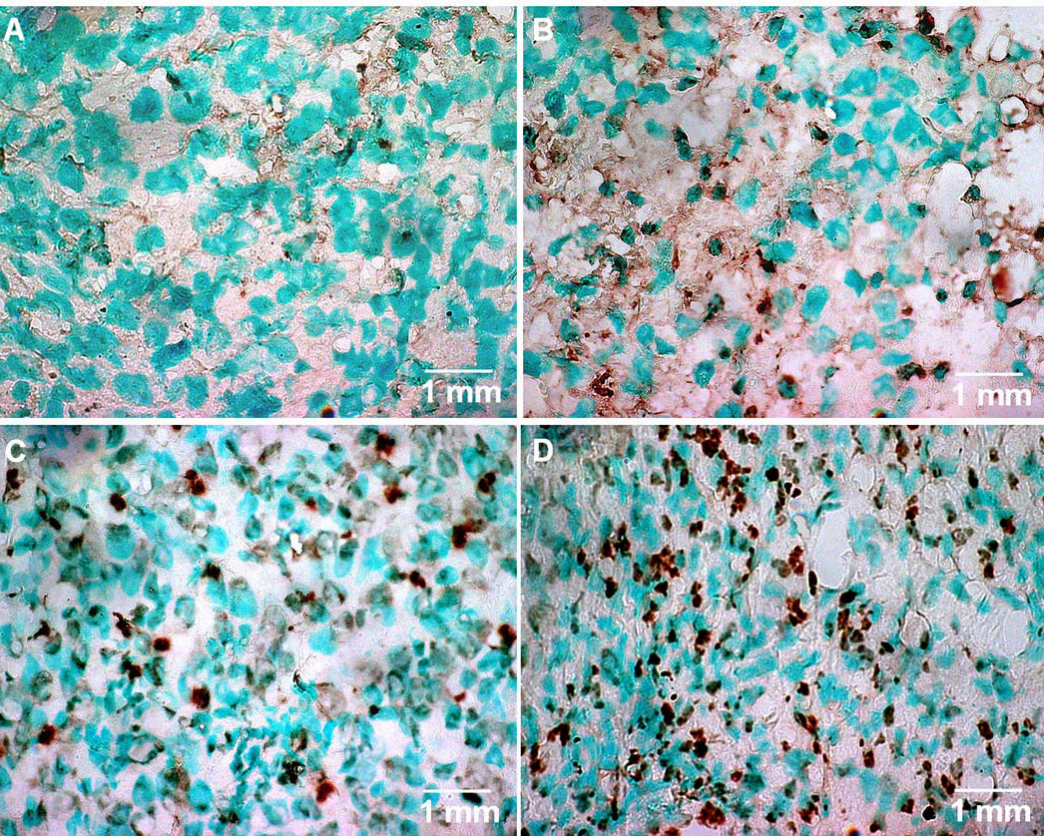
**The tunnel assay was performed on the tumors that were harvested from the animals at the end of the treatment**. Few isolated positive nuclei were noticed in (A) untreated tumors, (Apoptotic index (AI) - 6%). Both (B) PDT only (AI - 14%) and (C) Erbitux only (AI - 16%) treated tumors showed increased apoptosis compared to control. High levels of apoptotic nuclei were clearly exhibited by tumors treated with the (D) PDT plus Erbitux combination therapy (AI - 32%, *p *< 0.001). Magnification: 630×.

To determine whether the observed tumor growth suppression was caused by apoptotic cell death, a terminal deoxynucleotidyl transferase mediated dUTP nick-end-labeling (TUNEL) assay was performed (Figure 5). The tunnel assay was performed on the tumors that were harvested from the animals at the end of the treatment. Few isolated positive nuclei were noticed in untreated tumors (Apoptotic index (AI) - 6%). Both PDT only (AI - 14%) and Erbitux only (AI - 16%) treated tumors showed increased apoptosis compared to control. High levels of apoptotic nuclei were clearly exhibited by tumors treated with the PDT plus Erbitux combination therapy (AI - 32%, *p *< 0.001).

### EGFR phosphorylation

**Figure 6:**
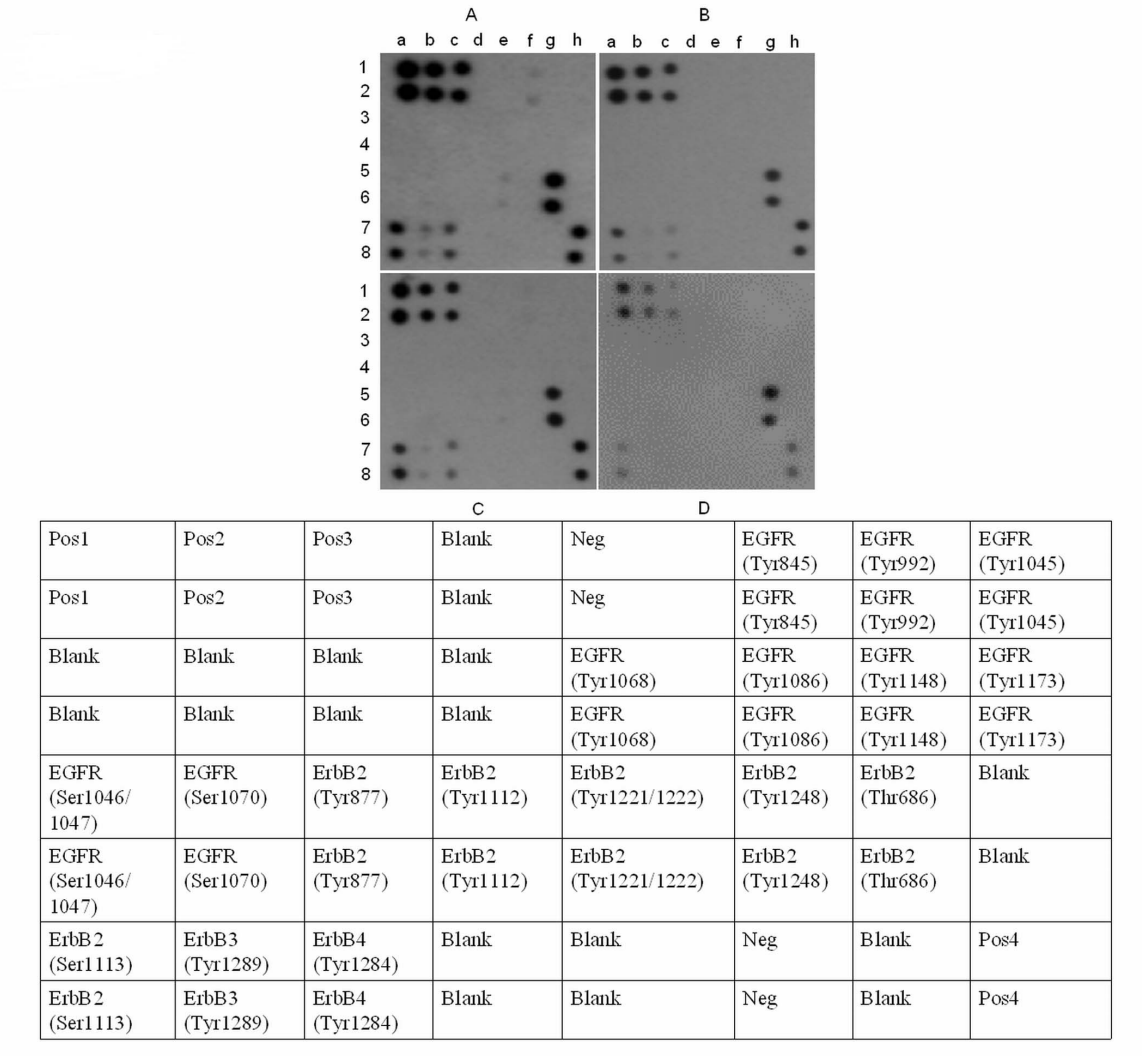
**Phosphorylation statuses of EGFR sites were determined using antibody arrays**. Increased phosphorylation of ErbB2(Thr686), ErbB2(Ser1113) and limited phosphorylation of EGFR(Thy845), ErbB2(Tyr1221/1222), ErbB3(Tyr1289) and ErbB4(Tyr1284) sites was seen in the control group. In the monotherapy groups, ErbB2(Thr686), (Ser113) and ErbB4(Tyr1284) sites were phosphorylated. Inhibition of most of the EGFR phosphorylation sites was observed in combination therapy groups except for ErbB2(Thr686) and (Ser1113). (A: Control, B: PDT, C: Erbitux and D: PDT + Erbitux).

To gain better understanding of the potential mechanisms of Erbitux and PDT treatments, we investigated the phosphorylation status of EGFR sites (Figure 6). Phosphorylation of EGFR can occur at different tyrosine sites that can lead to subsequent activation of different pathway. Increased phosphorylation of ErbB2(Thr686), ErbB2(Ser1113) and limited phosphorylation of EGFR(Thy845), ErbB2(Tyr1221/1222), ErbB3(Tyr1289) and ErbB4(Tyr1284) sites was seen in the control group. In the monotherapy groups, ErbB2(Thr686), (Ser113) and ErbB4(Tyr1284) sites were phosphorylated. Inhibition of most of the EGFR phosphorylation sites was observed in combination therapy groups except for ErbB2(Thr686) and (Ser1113). Though, phosphorylation at site Thr686 was greater than Ser1113.

### Expression of EGFR target genes

**Figure 7:**
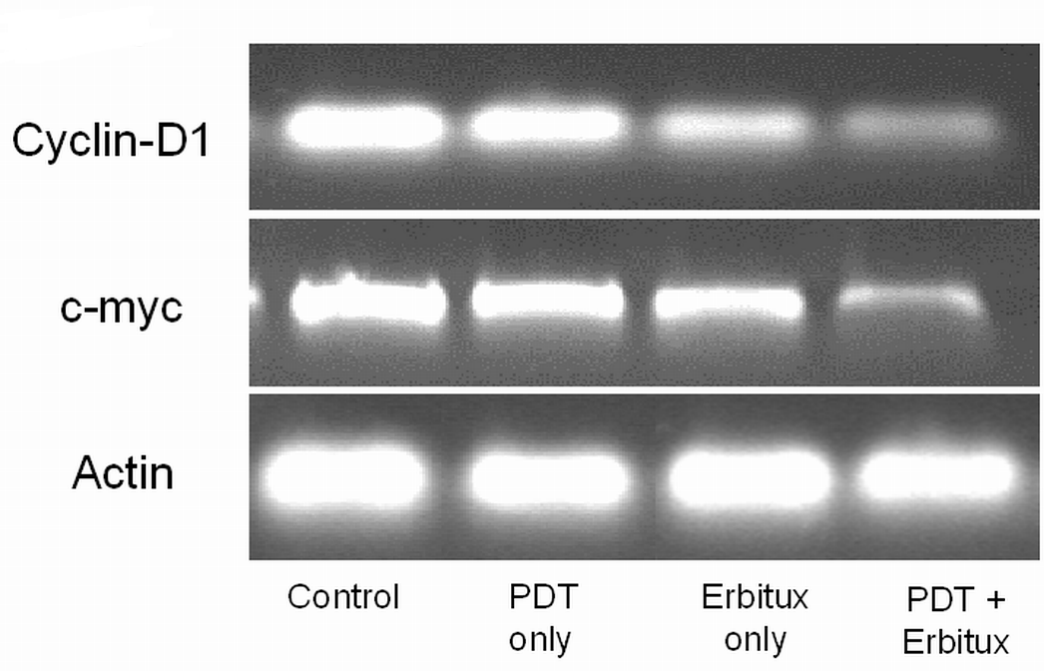
**The effect of EGFR inhibition on target genes cyclin-D1 and c-myc was evaluated at the RNA level**. Cyclin D1 is an important regulator of G1 to S-phase transition and overexpression of cyclin D1 has been linked to the development and progression of cancer. c-Myc is activated in a variety of tumor cells and plays an important role in cellular proliferation, differentiation, apoptosis and cell cycle progression. Downregulation of cyclin-D1 and c-myc was observed in the tumors treated with PDT and Erbitux (*p *< 0.05) when compared with the other groups.

The effect of EGFR inhibition on target genes cyclin-D1, c-myc was evaluated at the RNA level (figure 7). Cyclin D1 is an important regulator of G1 to S-phase transition and overexpression of cyclin D1 has been linked to the development and progression of cancer. c-myc is activated in a variety of tumor cells and plays an important role in cellular proliferation, differentiation, apoptosis and cell cycle progression. Downregulation of cyclin-D1 and c-myc was observed in the tumors treated with PDT and Erbitux (*p *< 0.05) when compared with the other groups.

## Discussion

PDT is being successfully used in clinics for the treatment of superficial lesions of both malignant and non-malignant diseases. However, treating solid tumors is still a challenge due to issues related to penetration of light, non-homogeneity and geometry of the tumors [21]. Triggering of angiogenesis is also dependent on different PDT parameters such as drug/light dosage and drug light interval. Previous studies have shown that sub-optimal PDT elicits increased angiogenesis [22,23]. In our earlier study we have reported that high dose light PDT with high fluence rate induces the overexpression of VEGF compared to low dose light PDT [24]. We have also noticed that predominantly cellular targeting long drug light interval PDT can induce greater expression of angiogenic proteins compared to vascular targeting short drug light interval PDT [25]. Therefore, there is a need for continued investigation to enhance the anti-tumor efficacy of PDT for improved response and expanded use. In this study, we evaluate the use of EGFR inhibitor Erbitux in combination with PDT to improve the tumor responsiveness in a bladder tumor xenograft model.

Bladder cancer treatment remains a challenge though significant progress has been made in the prevention of disease progression and the improvement of patient survival rates. PDT has been successfully used to treat recurrent or drug resistant superficial bladder cancer. 5-aminolevulinic acid (ALA)-PDT has shown to be an effective treatment option for patients with superficial bladder cancer [26]. However, ALA-PDT can cause pain and would require some form of local anesthesia. Some investigators have concluded that in most clinical trials of bladder cancer, the PDT treatment was overly aggressive and resulted in long lasting and severe urinary complications [27]. Nseyo et al. [28] suggested multiple treatments at lower drug and light doses to reduce the incidence and severity of symptoms following PDT of superficial bladder cancer. Single session whole bladder PDT using diffusion medium for isotropic light distribution was beneficial for patients treated with TCC refractory to traditional intravesical therapy [29]. However, patients with extensive flat papillary lesions did not appear to respond well. As can be seen, PDT treatment of bladder cancers continues to present major challenges and novel therapeutical approaches need to be explored.

Erbitux was approved by the US Food and Drug Administration (FDA) for use in combination with irinotecan for the treatment of metastatic colorectal cancer and it is also being used for the treatment of metastatic squamous cell carcinoma of the head and neck (SCCHN) [30,31]. Results of a large phase II study on irinotecan-refractory colorectal cancer patients have shown a significant response of 22.9% when Erbitux was combined with chemotherapy agent, irinotecan [32]. In another study, the response rate was significantly improved when Erbitux was combined with cisplatin in the first-line treatment of recurrent or metastatic SCCHN [33]. A randomized trial that compared radiotherapy plus Erbitux with radiotherapy alone in patients with stage III or IV non-metastatic SCCHN, demonstrated significantly longer locoregional control with radiotherapy plus Erbitux than with radiotherapy alone; moreover, progression-free survival were significantly longer and the overall response rate was significantly better with the combination therapy [34]. Recent results from a phase III randomised study demonstrated that the Erbitux given concomitantly with radiotherapy yields a significant clinical benefit over radiotherapy alone without any increase in radiotherapy-associated toxicity [35].

In our in vivo tumor regression study, we demonstrate that the combination therapy of Erbitux with PDT can improve the tumor response by attenuating the angiogenic process. A similar study conducted on a mouse model of human ovarian cancer in which C225 (Erbitux) was combined with PDT regimen produced synergistic reductions in mean tumor burden and significantly increased median survival [36]. In this study, PDT treated tumors did not exhibit significant tumor regression compared to combination therapy groups and this could be attributed to the high fluence rate that was administered during PDT. High fluence rate can deplete tumor oxygen to a large extent, thereby stimulating the production of stress induced survival molecules that reduce the effectiveness of PDT and affect tumor control [4]. More importantly, the administration of high light dose for this experiment was to test our hypothesis that combining PDT with Erbitux can improve tumor control and also to evaluate the effectiveness of Erbitux in reducing EGFR concentrations. Our investigations have indicated that Erbitux alone as monotherapy was not effective in controlling tumor growth. One of the possible reasons for this observation could be the fact that tumors overexpressing EGFR might not be sensitive to Erbitux. Although we would assume that tumors overexpressing EGFR would respond well to anti-EGFR therapy, studies have demonstrated that the level of EGFR expression does not have any impact on tumor response rates as a significant number of EGFR-positive tumors could be resistant to Erbitux [37,38]. The group that received the combination therapy of PDT and Erbitux exhibited accelerated growth a week after PDT which could be due to an increase in the expression of angiogenic growth factors either due to hypoxia, induced by oxygen depletion during PDT light irradiation or incomplete treatment. Our earlier results have shown increased expression of angiogenic growth factor VEGF at 72 h post PDT [39]. In this study, the regular administration of Erbitux after PDT treatment could have blocked the EGFR pathway and reduced angiogenesis. Therefore, our data supports the hypothesis that combination therapy of PDT and Erbitux would be more effective in preventing angiogenesis compared to monotherapy alone.

To further substantiate our results we performed western blotting, immunohistochemistry and immunofluorescence to determine the EGFR levels in all the treatment groups. EGFR immunoreactivity was localized mainly in the cell membranes and to a lower extent in the cytoplasm. It has been well established that the core of solid tumors is hypoxic, and that hypoxic tumor environment is sufficient to trigger EGFR expression in tumors [40]. Previous studies have reported the downregulation of EGFR after PDT [41,42]; in marked contrast our results demonstrated an increase in EGFR expression post hypericin-mediated PDT. This observation could be attributed to numerous reasons such as the light/drug dosage, the complexity of tumor microenvironment and the properties of the photosensitizer [4]. Combined antitumor activity of Erbitux with standard chemotherapy and radiotherapy is well documented in the treatment of different types of tumors and is reported to be more efficacious than individual monotherapies [19]. In this study, combination modality of PDT and Erbitux was effective in reducing the expression of EGFR and that could have lead to the regression of tumors in this group.

In the current study, we have also shown that PDT plus Erbitux increased apoptosis in the treated tumors compared to PDT only and inhibitor only monotherapies. Erbitux has been known to increase apoptosis in various tumor models by different mechanisms, including upregulation of pro-apoptotic Bax protein [43], decrease in the expression of anti-apoptotic molecule Bcl-2 [44] and the activation of pro-apoptotic caspases [45]. Hypericin-PDT is also known to induce apoptosis in a dose-dependent manner with higher doses leading to necrosis. Based on the lack of tumor inhibition in the monotherapy groups, it can be noted that tumors treated with PDT alone and Erbitux alone induced limited apoptosis in bladder carcinoma tumors. Therefore in this investigation, it was observed that the combination therapy significantly increased tumor cell apoptosis and inhibited tumor progression. Preclinically, many studies have shown that treatment with Erbitux in combination with radiotherapy or chemotherapy enhances apoptotic cell death than individual therapies. In a similar manner, PDT induced apoptosis, could have been enhanced by the combination of Erbitux to the treatment regime.

By using EGF phosphorylation antibody array membranes, we examined the relative level of phosphorylation of specific sites for human EGFR receptors. Interestingly, we noted the phosphorylation of Threonine 686 site of ErbB2 in all the groups. Studies have suggested that the dysregulation of cellular protein kinase C [46] and protein kinase A [47] activity could phosphorylate ErbB2 on Thr-686 for the activation and proliferation of tumor cells. However, our findings suggest that ErB2 on Thr-686 may not be essential for regulation of tumor proliferation, as tumor control was observed in the PDT + Erbitux treated group. Phosphorylation of EGFR tyrosine 845, only noticed in control tumors, is implicated in the stabilization of the activation loop, providing a binding surface for substrate proteins and is capable of regulating receptor function and tumor progression [48]. c-Src is known to be involved in the phosphorylation of EGFR at Tyr845 [49]. The major autophosphorylation sites of ErbB2 are Tyr1248 and Tyr1221/1222 that lead to Ras-Raf-MAP kinase signal transduction pathway [50]. In control tumors, ErbB2 was phosphorylated at tyrosine 1221/1222 and is associated with high tumor grade and with shorter disease-free survival and overall survival [51]. Similarly, ErbB4 is able to induce phosphorylation of phosphatidylinositol 3-kinase regulatory subunit which is a pro-survival protein that prevents apoptosis [52,53]. Our data suggests that dephosphorylation of ErbB4 tyrosine 1284 is critical for tumor regression in the dual treatment group.

EGFR-mediated Ras-Raf-MEK-ERK and PI3K-PTEN-AKT pathways plays an important role in transmission of signals from membrane receptors to downstream targets that regulate apoptosis, cell growth and angiogenesis. Components of these pathways include genes such as Ras, B-Raf, PI3K, PTEN and Akt that can be mutated or aberrantly expressed in human cancer. Though we did not investigate these genes, it should be noted that they could cause resistance to anti-EGFR therapy. Numerous studies have reported Kras mutations as a predictor of resistance to Erbitux therapy and are associated with poor prognosis in colorectal cancer [54] and non-small cell lung carcinoma [55]. In a similar way, Braf mutation is also known to cause resistance to anti-EGFR therapy in colorectal cancers [56] and primary lung adenocarcinomas [57]. Mutation of PTEN tumor suppressor gene in human cancer cells leads to activated EGFR downstream signaling including PI3-kinase/AKT and have been linked to resistance to anti-EGFR targeted therapies [58]. However, in this study we investigated the role of EGFR target genes cyclin D1 and c-myc that are involved in cell proliferation. Our RT-PCR results showed downregulation of cyclin D1 and c-myc in the tumors treated with the combination therapy. Amplification of cyclin D1, a key cell cycle regulatory protein, appears to be an important event in bladder cancer and is often associated with cell proliferation and poor prognosis in human tumors [59]. In our study, downregulation of EGFR also resulted in reduction of cyclin D1. This observation could be due to the administration of Erbitux, that is known to cause cell cycle arrest in the G(1)/G(0)-phase, and also increases the expression of cyclin-dependent kinase inhibitors [60]. c-myc, another EGFR target gene that can obstruct the induction of apoptosis in tumor cells and lead to uncontrolled cell growth was reduced in the PDT plus Erbitux treated tumors. Over-expression and amplification of c-myc can play an important role in metastatic progression that indicates poor prognosis in different cancers [61]. These results suggest that EGFR target genes could play a role in tumor inhibition in bladder cancer by arresting cell cycle growth and inducing apoptosis.

## Conclusion

In conclusion, combination treatment of PDT and Erbitux can improve the tumor response of bladder carcinoma xenografts. In this study, we observed that PDT induced tumor destruction can be maintained and significantly enhanced by the administration of Erbitux. As PDT treated tumors have been shown to adapt to inflammation and vascular shutdown, and PDT alone may not be sufficient for effective treatment, there is a need for combination of different modalities to obtain better tumor response. The challenge is to choose the appropriate anti-angiogenesis agent in combination with optimal PDT dosimetry for potential clinical application.

## Methods

### Photosensitizer

A stock solution of 5 mg/ml hypericin (Molecular Probes, USA) was prepared by adding 200 μl of dimethyl sulfoxide, DMSO (Sigma Aldrich Inc, St Louis Mo, USA) to 1 mg of hypericin. The stock solution was further diluted in DMSO and PBS (1:3 v/v) and injected intravenously into the tail vein based on the weight of the animal at a dosage of 5 mg/kg.

### Dosage of Erbitux (Cetuximab)

Erbitux (Imclone) at a concentration of 2 mg/mL was administered intraperitonially at a dosage of 10 mg/kg.

### Cell culture and xenograft tumor model

MGH bladder cancer cells were cultured as a monolayer in RPMI-1640 medium supplemented with 10% fetal bovine serum, 1% non-essential amino acids (Gibco, USA), 1% sodium pyruvate (Gibco, USA), 100 units/ml penicillin/streptomycin (Gibco, USA) and incubated at 37°C, 95% humidity and 5% CO_2_. Before inoculation, the cell layer was washed with PBS, trypsinized and counted using a hemocytometer. Male Balb/c nude mice, 6-8 weeks of age, weighing an average of 24-25 g were obtained from the Animal Resource Centre (ARC), Western Australia. Approximately 3.0 × 10^6 ^MGH human bladder carcinoma cells suspended in 150 μl of Hanks' balanced salt solution (Gibco, USA) was injected subcutaneously into the lower flanks of the mice. The tumors were allowed to grow to sizes of 80 to 100 mm^3 ^in volume before PDT treatment was carried out and the tumors were measured three times a week.

### In vivo treatment protocol

The mice were randomized into 4 groups (10 animals per group) i.e., (i) Control (mice with untreated tumors), (ii) PDT only (iii) Erbitux only and (iv) PDT plus Erbitux. Treatment involved the intravenous injection of hypericin followed by irradiation with a light source consisting of filtered halogen light (Zeiss KL1500) fitted with a customized 560-640 nm band-pass filter. Light irradiation was performed 6 h post hypericin administration. A light dosage with fluence of 120 J/cm^2 ^and fluence rate of 100 mW/cm^2 ^was used for PDT treatment. Erbitux was administered by intraperitoneal injections (10 mg/kg) at time 0 (immediately after light exposure), 24 h, 48 h and then every other day up to 90 days post PDT. The mice were euthanized when either the tumor reached the 2-cm^3 ^ethical limit or at the end of the 90 day monitoring period. The tumors were harvested and divided into a few sections for immunohistochemistry, immunofluorescence, protein and RNA extraction. All procedures were approved by the Institutional Animal Care and Use Committee (IACUC), SingHealth, Singapore, and performed in accordance with international standards.

### Immunoblotting

Tissue lysate buffer (T-PER, Pierce, USA) along with protease inhibitor (Complete Mini, Roche, Germany) was added to the tumor that was crushed into powder in liquid nitrogen. Tissue and cell debris was removed by centrifugation and the lysate was stored at -80°C until use. Protein estimation of tumor lysates was performed using biorad protein assay solution and was quantified using the GeneQuant pro machine (Biochrom, UK). Following the addition of sample buffer to the lysates, 50 μg of protein was resolved onto SDS gel and transferred to nitrocellulose membrane (Pall Corporation, USA) using a TRIS/glycine/SDS electrode tank buffer, run for 2 h. Membranes were blocked overnight with 5% low fat milk powder-TBS-Tween and then washed thoroughly before probing with the primary antibody 1: 500 (EGFR, Cell Signaling. USA). After washing with TBS-Tween the membranes were incubated with HRP-linked secondary antibody (1:1000) for 1 h. The level of specific protein was visualized by chemiluminescence (Supersignal, Pierce Techonology, USA). The membrane was then exposed to X-ray film (Hyperfilm ECL, Amersham Biosciences, UK) and the signal was detected using film developer (Kodak M35, OMAT Processor, USA). The intensities of the signal were quantified by densitometer (Syngene, USA) and analysed with GeneTool (Syngene, USA).

### Immunohistochemistry

Processing of the samples was done using tissue processor (Leica TP 1020, Germany). Briefly the tissue samples were fixed in 10% formalin for 24 h, and then processed in an ascending series of ethanol and subsequently cleared with xylene and embedded in paraffin. The paraffin embedded bladder samples were sectioned at a thickness of 4 μm using a microtome (Leica RM 2135, Germany). The sections were mounted on superfrost/plus slides (Fischer Scientific, USA) and air-dried. On the day of staining the slides were heated in 60°C oven for 1 h and immersed in zylene for 10 min before rehydration in ethanol series. Sections were incubated in hydrogen peroxide for 10 min to block endogenous peroxidase activity. After which, the sections were incubated with EGFR (BD Biosciences Pharmingen, USA) primary antibody (1:100) for 1 h. To confirm the specificity of binding, normal mouse serum IgG_1 _(1:500) was used as negative control instead of primary antibody. Following extensive washing, sections were incubated for 30 min in the secondary biotinylated antibody followed by DAB Chromogen (Dako REAL EnVision Detection System, USA) for 10 min. Sections were then counter-stained with Harris's hematoxylin and dehydrated in ascending grades of ethanol before clearing in xylene and mounting under a cover slip. Images were captured using image processing software (AxioVision v 4.6.3.0, Carl Zeiss Imaging Solutions, GmbH, Germany). The images were saved in TIFF format and NIH Image J v1.62 software was used to analyze and quantify the expression of EGFR. Briefly, the percentage of positively stained cells was calculated by obtaining the area of the immunostained regions divided by the area of the total image. EGFR scoring was performed based on the prevalence of tumor cell membrane staining (EGFR score 1 = weak intensity and incomplete staining of less than 10% of tumor cells; score 2 = moderate and complete staining of between 11 to 20% of tumor cells; score 3 = strong and complete staining of more than 21 to 30% of tumor cells.)

### Immunofluorescence

Fresh frozen tissue sections were fixed with 4% paraformaldehyde for 2 min. The specimen was blocked for 1 h with normal goat serum in Triton X-100. After blocking, sections were incubated overnight with EGFR primary antibody (Cell Signaling, US) at 4°C. Nonimmune IgG was used as control. After rinsing in PBS, the specimen was stained with FITC-conjugated secondary antibody for 2 h at room temperature in dark. Slides were then rinsed with PBS and stained with DAPI for 30 min. Finally, the slides were rinsed and mounted with Vectashield^® ^Mounting Medium (Vector Laboratories Inc., USA). Immunofluorescence images were captured using a laser confocal fluorescence microscope (Meta LSM 510, Carl Zeiss, Germany) and image analysis was performed using the ImageJ software (1.41o, W. Rasband, National Institute of Health, USA).

### TUNEL assay for DNA fragmentation

Apoptosis was assessed by using the DNA fragmentation detection kit, TdT- FragEL™ (Oncogene Research Products, USA). Briefly, 15 μm tissue cryosections were fixed with 4% formaldehyde for 15 min. The slides were then rinsed in 1× TBS and permeabilised with 20 μg/ml proteinase K for 10 min at room temperature. A positive control was generated by adding 1 μg/μl DNase I in 1× TBS/1 mM MgSO_4_. Reaction mixture (60 μl) that included 57 μl TdT Labeling reaction mix and 3 μl TdT enzyme was added to the sections and left for 1.5 h at 37°C. After rinsing, the specimens were incubated with HRP conjugate for 30 min. Finally DAB solution was added to the sections to generate an insoluble colored product at the site of DNA fragmentation and later counterstained with methyl green. The TUNEL-stained sections were then examined under light microscopy to determine the apoptotic indices. The apoptotic index (AI) was defined as the percentage of apoptotic nuclei counted per 1000 neoplastic nuclei; fields were chosen randomly at 630× magnification.

### EGFR phosphorylation

A human EGFR phosphorylation antibody array (RayBioTech, USA) was used to simultaneously detect phosphorylation of 17 different sites for Human EGFR in cell lysates. The cell lysates were prepared from MGH bladder cancer cells that were treated with PDT, Erbitux alone, PDT plus Erbitux and control. The components in the kit included array membranes, biotin-conjugated anti-cytokines, HRP-conjugated streptavidin and detection buffer. The manufacturer's protocol was followed to perform the experiments. Briefly, the antibody array membranes were treated with blocking buffer, after which the sample (cell lysate) was added to the membranes and incubated for 2 h. After extensive washing the membranes were incubated with cocktail of biotin-conjugated anti-EGFR was used to detect phosphorylated EGFR on activated receptors. After incubation with HRP-streptavidin, the signals were visualized using chemiluminescence. The membranes were exposed to X-ray film (Hyperfilm ECL, Amersham Biosciences, UK) and signal was detected using a film developer (Kodak M35, OMAT Processor, USA). The intensities of the signal were quantified by densitometer (Syngene, USA). By comparing the intensity of signals the relative expression levels of the phosphorylated EGFR sites were determined. Positive control was used to normalize the results from different membranes being compared.

### RNA isolation

Total RNA was extracted from tumor tissue using the commercially available Nucleospin RNA II kit (Macherey-Nagel, Germany). Briefly, the frozen tissue samples were crushed into powder using liquid nitrogen and lysis buffer, and β-mercaptoethanol was added to prepare the lysate. The lysate was then filtered and 70% ethanol was added to adjust RNA binding to the columns. Later DNA digestion was performed and pure RNA was eluted. RNA quality and purity was checked using UV Spectrophotometry and by detecting the ribosomal RNA integrity.

### RT-PCR analysis of gene expression

RT-PCR was performed using the Qiagen OneStep RT-PCR kit. Briefly, a 50 μl final volume containing 10 μl 5× QIAGEN OneStep RT-PCR buffer, 2 μl dNTP Mix, 2 μl QIAGEN OneStep RT-PCR enzyme mix, 1 μl of RNase inhibitor, 1.5 μl of forward and reverse primers (at a final concentration of 0.6 μM) and RNase free water was used to perform the reaction. Reverse transcription and PCR was carried out sequentially in the same tube. The resulting mixture was heated at 50°C for 30 min, the initial PCR activation step was performed for 15 min at 95°C, 3-step cycling of denaturation for 1 min for 94°C, annealing for 1 min at 50-68°C and extension for 1 min at 72°C and 25 cycles was carried out. The final extension was performed for 10 min at 72°C. Primers were commercially synthesized by Sigma Aldrich. After RT-PCR, 20 μl of individual RT-PCR product and 2 μl 6× loading buffer (Fermantas, USA) was electrophoresed in 1.5% agarose gel in TAE buffer. The gel was viewed and captured as a digital image by the Gel Documentation System (Bio-Rad Laboratories). Semi-quantitative measurements were derived by expressing the RT-PCR fragment's band intensity ratio with gene of interest against actin. The primer sequences and GenBank accession numbers are as follows: Cyclin D1 (GenBank No. NM_007631) forward: 5'GGTGCTTGGGAAGTTGTGTT3' and reverse: 5'CTCCGTCTTTGTGGTTTGGT3' and c-myc (GenBank No. NM_012333.3) forward: 5'TTACAAAGCCGCCGACTC3' and reverse 5'CTGCACCAAGGAATAGCTCC3'. β-Actin (GenBank No. NM_001101.2) forward: 5'TGTCACCAACTGGGACGATA3' reverse: 5'TCTCAGCTGTGGTGGTGAAG3'.

### Statistical analysis

Tumor volume was calculated by using the formula, volume = (π/6 × d1 × d2 × d3), where d1, d2 and d3 are tumor dimensions in 3 orthogonal directions. The effectiveness of the treatment in terms of tumor growth inhibition was evaluated on day 29 when tumor volumes reached maximum size in the control group. This was calculated by determining the percentage difference in tumor growth volumes for the treatment groups compared to control tumor volume. One-way analysis of variance with the Bonferroni correction was performed to analyze the data obtained in this study using Prism 3.0 software (Graphpad Prism, San Diego, CA). A *P *value of < 0.05 was considered to be significant.

## Abbreviations list

PDT: photodynamic therapy; EGFR: epidermal growth factor receptor; FDA: Food and Drug Administration; SCCHN: squamous cell carcinoma of the head and neck; MGH: Massachusetts General Hospital; IHC: immunohistochemistry; IF: immunofluorescence; TUNEL: terminal deoxynucleotidyl transferase mediated dUTP nick-end-labeling; AI: Apoptotic index; Thr: threonine; Tyr: Tyrosine; Ser: Serine; DMSO: dimethyl sulfoxide; PBS: phosphate-buffered saline; ARC: Animal Resource Centre; IACUC: Institutional Animal Care and Use Committee.

## Competing interests

The authors declare that they have no competing interests.

## Authors' contributions

RB: designed, carried out the experiments, analyzed data and drafted the manuscript. YYG: supervised the project and commented on the manuscript. KCS: supervised the project and commented on the manuscript. MO: provided funding, supervised the project and corrected final manuscript. All authors read and approved the final manuscript.
